# Ferritin levels and risk of metabolic syndrome: meta-analysis of observational studies

**DOI:** 10.1186/1471-2458-14-483

**Published:** 2014-05-21

**Authors:** Victoria Abril-Ulloa, Gemma Flores-Mateo, Rosa Solà-Alberich, Begoña Manuel-y-Keenoy, Victoria Arija

**Affiliations:** 1 Faculty of Medicine and Health Sciences, Universitat Rovira i Virgili, Reus, Spain; 2 Dirección de Investigación (DIUC), Universidad de Cuenca, Cuenca, Azuay, Ecuador; 3 Unitat de Suport a la Recerca Tarragona-Reus, Institut Universitari d’Investigació en Atenció Primària Jordi Gol (IDIAP Jordi Gol), Tarragona, Spain; 4 CIBERobn Physiopathology of Obesity and Nutrition, Institute of Health Carlos III (ISCIII), Madrid, Spain; 5 Institut d’Investigació Sanitària Pere Virgili (IISPV), Reus, Spain; 6 University of Antwerp, Antwerp, Belgium; 7 Nutrition and Public Health Unit, Universitat Rovira i Virgili, C/Sant Llorenç 21, 43201 Reus, Spain

**Keywords:** Ferritin, Metabolic syndrome, Meta-analysis

## Abstract

**Background:**

Elevated ferritin levels have been associated with single cardiovascular risk factors but the relationship to the presence of metabolic syndrome is inconclusive.

The aim of this systematic review and meta-analysis of published observational studies was to estimate the association between serum ferritin levels and metabolic syndrome in adults.

**Methods:**

The Pubmed, SCOPUS and the Cochrane Library databases were searched for epidemiological studies that assessed the association between ferritin levels and metabolic syndrome and were published before September 2013. There were no language restrictions. Two investigators independently selected eligible studies. Measures of association were pooled by using an inverse-variance weighted random-effects model. The heterogeneity among studies was examined using the *I*^2^ index. Publication bias was evaluated using the funnel plot.

**Results:**

Twelve cross-sectional, one case–control and two prospective studies met our inclusion criteria including data from a total of 56,053 participants. The pooled odds ratio (OR) for the metabolic syndrome comparing the highest and lowest category of ferritin levels was 1.73 (95% CI: 1.54, 1.95; *I*^2^ = 75,4%). Subgroup analyses indicate that pooled OR was 1.92 (95% CI: 1.61, 2.30; *I*^2^ = 78%) for studies adjusting for C-reactive protein (CRP), and 1.52 (95% CI:1. 36, 1.69; *I*^2^ = 41%) for studies that did not adjust for CRP (*P* = 0.044). This finding was remarkably robust in the sensitivity analysis. We did not find publication bias.

**Conclusions:**

The meta-analysis suggests that increased ferritin levels are independently and positively associated with the presence of the metabolic syndrome with an odds ratio higher than 1.73.

## Background

The metabolic syndrome, currently prevalent in 20% - 25% of the world's adult population, is a significant risk factor for cardiovascular disease, type 2 diabetes and cancer [1]. It consists of clinical symptoms and abnormal lab results, including abdominal obesity, insulin resistance, hyperglycemia, hyperlipidemia, and hypertension [1].

Ferritin, an ubiquitous intracellular protein that is key in the regulation of iron homeostasis, is an accepted biomarker to evaluate body iron stores [2]. However, increasing evidence indicates that elevated body iron stores may be associated with adverse health outcomes. Elevated serum ferritin levels have been demonstrated to independently predict type 2 diabetes mellitus in several meta-analyses [3-5]. In cross-sectional studies, elevated ferritin levels have been associated with hypertension [6], dyslipidemia [7,8], elevated fasting insulin and blood glucose levels [9], and central adiposity [10]. However, no meta-analysis has specifically focused on high ferritin blood concentrations in relation to the presence of the metabolic syndrome. Moreover, the presence of inflammation has not been systematically taken into account, leading to conflicting results [11-22].

Evaluating if high serum ferritin is associated with the metabolic syndrome is relevant for both the clinician and the public health areas that focus on screening and prevention.

In order to address this issue we aimed in this study to meta-analyze the findings of published original research articles investigating the relationship between serum ferritin and the presence of the metabolic syndrome in adults of both genders in prospective cohort and cross-sectional studies.

## Methods

### Search strategy

We searched PubMed (http://www.ncbi.nlm.nih.gov/pubmed), the SCOPUS and Cochrane Central Database for observational studies, investigating the association between serum ferritin levels and metabolic syndrome.

We used free text and the Medical Subject Heading (MeSH) terms *metabolic syndrome, iron, ferritin, transferrin, ferritins, iron stores, iron status, iron intake, iron consumption, heme iron*. The search period was all-inclusive until February 2014; no language restrictions were added. We also reviewed the reference lists of the retrieved original articles.

### Study selection

We included all observational studies (cross-sectional, case–control and prospective) that were conducted in adults aged ≥ 18 years, assessing the association between serum ferritin and metabolic syndrome.

Exclusion criteria were the following: 1) no original research (reviews, editorials, non-research letters); 2) case reports and case series; 3) studies concerning children, adolescents and pregnant women; 4) study subjects with hemochromatosis, chronic liver disease, liver cirrhosis or chronic renal diseases; and 5) studies with Type 1 diabetes mellitus or Type 2 diabetes mellitus participants.

For study populations generating more than one report [20,22-26], we selected the study with the largest number of participants [20,22,27].

### Data extraction and quality assessment

Two investigators (G.F-M and V.A-U) independently reviewed the search results and selected articles to determine eligibility and to extract study data. A third investigator independently reviewed the published data (V.A). They resolved discrepancies by consensus. Extracted data included information on the study design (prospective cohort, cross-sectional and other designs), measures of association used (odds ratio or hazard ratio), country of origin, population, sex, average age of participants, number of participants, ferritin assay technique, ferritin levels, metabolic syndrome criteria and outcomes.

The investigators of the original studies were contacted if relevant information on eligibility or key study data were not available in the published report.

To assess study quality, we used the STROBE statement of observational studies [27]. Each of the criteria was categorized as clearly yes or clearly no. A score between 0 and 22 was assigned to allow for quality analysis (0 denoted noncompliance with any criteria, and 22 denoted fulfillment of all criteria). Our meta-analysis was registered on the website of the International prospective register of systematic reviews, PROSPERO (CRD42012002258).

### Data synthesis and statistical analysis

Measures of association (odds ratio (OR), relative risks or hazard ratios) and their 95% CIs were extracted or derived by using the data reported in the publications. When several measures of association were reported, we chose the measure obtained from the model of the highest category for ferritin concentration and as second choice, the measure adjusted for most covariates. For studies that categorized ferritin levels, we compared the risk of metabolic syndrome in the highest with the lowest ferritin category. For studies reporting only mean levels of ferritin in case and non-case subjects [12,14], we used linear discriminant function methods [28] to calculate the OR in a comparison of the 75th to the 25th percentiles of the ferritin distribution in non-case subjects, assuming a normal distribution for ferritin.

To pool OR estimates from individual studies, we used an inverse variance weighted random-effects model. Heterogeneity was quantified with the *I*^2^ statistic which describes the proportion of total variation in the study estimates that is due to heterogeneity [29]. To explore sources of heterogeneity, we performed subgroup analysis and meta-regression to evaluate whether results were different depending on the study design (prospective (cohort), cross-sectional and other designs, on the measure of association used (odds ratio or hazard ratio), geographic area (Asian, Europe or American), adjusted for C-reactive protein (CRP) (yes or not), quality of the study (<30 points or equal or higher than 30 points of SCORE statement of 34 as maximum points), ferritin technique assay (immunoradiometric assay RIA; immunoturbidimetric assay, TIA; or others) and study size (<300 or equal or higher than 300 participants).

We used sensitivity analyses to assess the relative influence of each study on the pooled estimates by omitting one study at a time. Finally, we assessed publication bias using funnel plots [30].

Our results were expressed as pooled OR.

Statistical analysis was performed using Stata software (version 11.0; Stata Corp, College Station, TX, USA).

## Results

### Study selection

**Figure 1:**
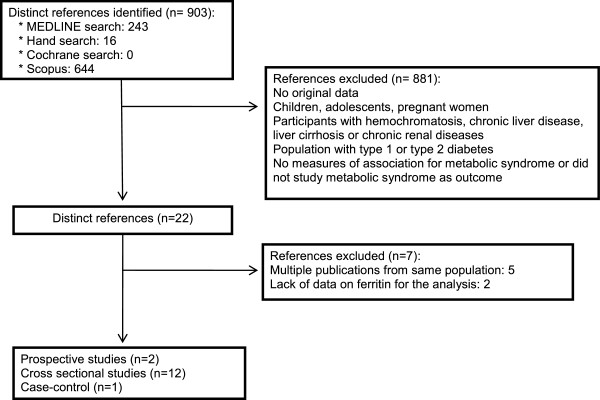
Flow diagram of the study selection process.

The search strategy retrieved 243 unique citations in the Pubmed, 644 in the SCOPUS and 0 in the Cochrane Library (Figure 1). Of these citations, 238 and 636 respectively, were excluded after screening on the basis of title and abstract and 7 after full-text review, leaving twelve cross sectional studies [11-20,22,31], one case–control study [21] and two prospective cohort studies [26,32] for final inclusion in the meta-analysis. Fourteen articles were in English and one in Korean (Its abstract was in English). The fifteen studies found [11-22,26,31,32] were published between 2004 and February 2014. One study was performed in the United States [11], nine studies in Asia [13,15-18,20,22,26,31]; four in Europe [12,14,21,32] and one in Chile [19]. The number of subjects per study varied between 155 [19] to 13,084 [26] (Table 1).

**Table 1:** Cross sectional and cohort studies on Ferritin level and of the Metabolic Syndrome

**First author Year (Reference number)**	**Country**	**Study design**	**Population**	**Men (%)**	**Age years**	**Sample**	**Ferritin assay**	**Metabolic syndrome criteria**	**Ferritin concentration (μg/l)**		*Quality score
									**Men**	**Women**	
Jehn, 2004 [11]	U.S.	Cross-sectional	NHANES III (1988–1994) U.S. Population	48	48	5,949	RIA	NCEP ATPIII	Q1:62^a^ Q4:318^a^	Premenopausal: Q1:11^a^ Q4:89^a^ Postmenopausal: Q1:35^a^ Q4:212^a^	30
Bozzini, 2005 [12]	Italy	Cross-sectional	Verona Heart Project	73	58	479	NIA	NCEP ATPII	Both genders: With MS: 124 (111–138)^b^ Without MS: 83 (73–94)^b^		19
Choi, 2005 [13]	Korea	Cross-sectional	Welfare Centers of Seoul Metropolitan	0	72	959	EIA	NCEP original		With MS: 74 ± 2^c^ Without MS: 59 ± 2^c^	28
Soto González 2006 [14]	Spain	Cross-sectional	Patients of the Endocrinoloy and Nutrition Service of Hospital	34	38	598	RIA	NCEP ATPIII	Both genders: With MS: 133.9 ± 141.1^d^ Without MS: 66.8 ± 71.8^d^		31
Vari, 2007 [32]	France	Prospective cohort	DESIR cohort French people 6 years of follow up	49	47	944	NIA	NCEP ATPIII	At baseline 178 ± 90^d^	At baseline Premenopausal women: 56 ± 40^d^ Postmenopausal women: 92 ± 54^d^	32
Shi, 2008 [15]	China	Cross-sectional	The 2002 National Nutrition and Health survey	46	40-49	2,816	RIS	NR	Q4:257 (176–500)^e^	Q4:170 (96–504)^e^	30
Sun, 2008 [16]	China	Cross-sectional	Nutrition and Health of Aging population	44	58	3,165	TIA	NCEP ATPIII	Q1:71 (68–73.2)^b^ Q4:327 (316–340)^b^	Q1:52 (51–54)^b^ Q4:231(224–239)^b^	30
Ryu, 2008 [17]	Korea	Cross-sectional	Korean Rural GENOMIC Cohort	38	58	1,444	N.R	NCEP ATPIII	Q1:45 ± 2^f^ Q4:258 ± 12^f^	Q1:17 ± 2^f^ Q4:131 ± 1^f^	32
Kim, 2011 [18]	Korea	Cross-sectional	Healthy volunteers	53	51	7,253	TIA	NCEP ATPIII	176 ± 108^d^	75 ± 55^d^	30
Park, 2012 [26]	Korea	Prospective cohort	Check-up of men in Health Promotion Center	100	44	13,084		International Diabetes Federation	At baseline 112 ± 64^d^	-------------	31
Kang, 2012 [20]	Korea	Cross-sectional	South Korean general population KNANHES IV (2007–2008)	44	48	7,346	RIA	NCEP ATPIII	Q1:46 (33, 55)^g^ Q4:194(167, 247)^g^	Q1:13 [9,17]^g^ Q4:90 (75, 114)^g^	27
Hämäläinen, 2012 [21]	Finland	Case–control	People invited to heath check up in 2004	45	52	766	EIA	NCEP ATPIII	With MS: 216 ± 165^d^ Without MS: 151 ± 112^d^	With MS: 94 ± 75^d^ Without MS: 61 ± 48^d^	28
Leiva, 2013 [19]	Chile	Cross-sectional	Research program of Risk Factors for Cardiovascular Disease of Talca	31	57	155	EIA	NCEP ATPIII	With MS: 72 (47–112)^h^ Without MS: 55 (36–96)^h^	With MS: 54 (34–85)^h^ Without MS: 27 (13–60)^h^	30
Chang, 2013 [22]	Taiwan	Cross-sectional	Third national nutritional and health survey in Taiwan (NAHSIT 2005-2008)	43	55	2,654	EIA	NCEP ATPIII modified	229 ± 349^d^	119 ± 180^d^	27
Li, 2013 [31]	China	Cross-sectional	China Health and Nutrition Survey	47	51	8,441	RIA	NCEP ATPIII modified	Q1:52^a^ Q4:423^a^	Q1:12.9^a^ Q4:142.7^a^	30

EIA, Electrochemiluminescence immunoassay; NIA, Nephelometric immunoassay; NR, not reported; RIA, immunoradiometric assay; TIA, immunoturbidimetric assay. *Quality score of STROBE Statement.

^a^Median of quartile; ^b^Geometric mean (95% CI); ^c^Geometric mean ± SD; ^d^Mean ± SD; ^e^Mean of quartile (minimum-maximum); ^f^Geometric mean ± SE; ^g^Median (range); ^h^Geometric mean (range).

SD: standard deviation; SE: standard error; CI: confidence interval; MS: metabolic syndrome; Q1: quartile 1; Q4: quartile 4: T1: tertile 1; T3: tertile 3.

The STROBE quality score of studies included 7 had 30 points or more while 5 studies had <30 points.

The metabolic syndrome was defined according with the criteria by the National Cholesterol Education Program Adult Treatment Panel III criteria (NCEP ATP III) [33] in all studies except one defined by International Diabetes Federation Task Force on Epidemiology and Prevention [26].

### Meta-analysis of serum ferritin levels and metabolic syndrome

**Figure 2:**
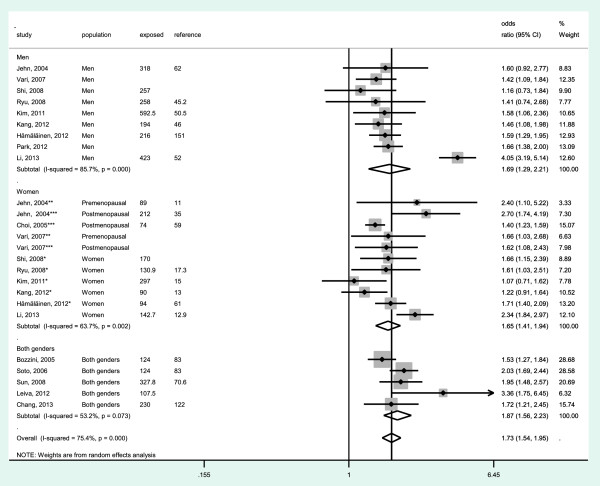
**Meta-analysis of the association of ferritin levels with metabolic syndrome in observational studies.** Studies are divided by gender (men, women and both genders). Odds ratios correspond to comparisons of extreme categories of exposure within each study. The area of each square is proportional to the inverse of the variance of the log odds ratio. Horizontal lines represent 95% CI. Diamonds represent pooled estimates from inverse-variance weighted random-effects models. NR, not reported.

Data from a total of 56,035 participants were analyzed. The pooled OR when comparing the highest to the lowest category of ferritin levels and metabolic syndrome was 1.73 (95% CI: 1.54, 1.95); heterogeneity: *P* < 0.001; *I*^2^ = 75,4% (Figure 2). The pooled OR for men was 1.69 (95% CI: 1.29, 2.21); heterogeneity: *P* < 0.001; *I*^2^ = 87,7%; for women it was 1.65 (95% CI: 1.41, 1.94); heterogeneity: *P* = 0.002; *I*^2^ = 63.7% and for studies that included both genders it was 1.87 (95% CI: 1.56, 2.23); heterogeneity: *P* = 0.073; *I*^2^ = 53.2%.

Meta-regression and subgroup analysis showed that adjusting by CRP and quality of studies significantly influenced the pooled estimates (*P* = 0.044 and *P* = 0.038 respectively) (Table 2). An stronger association between ferritin levels and metabolic syndrome was detected in studies adjusted by CRP [OR = 1.92 (95% CI: 1.61, 2.30; *I*^2^ = 78%)] compared to studies that did not adjust by CRP [OR = 1.52 (95% CI: 1.36, 1.69; *I*^2^ = 41%)]. Moreover, studies with high quality (≥30 points) showed a stronger association between serum ferritin levels and metabolic syndrome compared to studies with medium-poor quality (<30 points) (OR = 1.87 vs OR = 1.43).

**Table 2:** Stratified odds ratio for metabolic syndrome

**Subgroup**	**Number of studies**	**Odds ratio (95% CI)**	***I***^**2**^	***P*-value**
**Study design**				
Prospective cohort	2	1.59 (1.38, 1.82)	0%	
Cross-sectional	12	1.78 (1.51, 2.09)	81%	
Case–control study	1	1.65( 1.43, 1.91)	0%	0.551
**Measure of association**				
Odds Ratio	13	1.62 (1.47, 1.79)	47%	
Hazard Ratio	2	1.61 (1.49, 1.75)	37%	0.520
**Geographic area**				
Asian	9	1.67 (1.38, 2.02)	84%	
Europe	4	1.66 (1.50, 1.83)	14%	
American	2	2.41 (1.77, 3.27)	11%	0.239
**Adjusted for CRP**				
Yes	9	1.92 (1.61, 2.30)	78%	
No	6	1.52 (1.36, 1.69)	41%	0.044
**Quality of SCORE statement**				
<30 points	4	1.43 (1.27, 1.61)	0%	
≥30 points	11	1.87 (1.62, 2.17)	79%	0.038
**Ferritin assay**				
RIA	5	1.93 (1.49, 2.51)	85%	
TIA	2	1.53 (1.08, 2.16)	64%	
Others	6	1.56 (1.42, 1.70)	17%	0.091
**Study size, population**				
<300	4	1.76 (1.512, 2.06)	57%	
≥300	11	1.70 (1.46, 1.98)	78%	0.152

CRP, C-reactive protein; RIA, immunoradiometric assay; TIA, immunoturbidimetric assay.

Other sources of heterogeneity investigated, such as study design (*P* = 0.551), measure of association (*P* = 0.520), geographic area (*P* = 0.239), ferritin assay technique (*P* = 0.091), study size (*P* = 0.152), did not influence pooled estimates (Table 2).

In sensitivity analyses, the exclusion of individual studies did not modify the estimates substantially and the pooled odds ratio ranged from 1.64 to 1.76.

## Discussion

The present study is the first meta-analysis summarizing the independent positive association between ferritin levels and the metabolic syndrome. The highest category of ferritin levels was independently associated with a 1.73 higher presence of metabolic syndrome when compared to the lowest category. This association was stronger when adjusted for an inflammatory biomarker such as CRP levels. The initial results of the meta-analysis were consistent when tested for sensitivity analyses.

The positive independent association between serum ferritin levels and presence of metabolic syndrome is biologically plausible. Iron is an essential trace element for the human body, involved in cellular processes and a key component of various enzymes. It can also be toxic due to oxidative stress generation by the Fenton reaction, causing organic biomolecular oxidation [34]. This process is at the basis of pathologies like diabetes mellitus, neoplasia and degenerative brain disorders [34,35].

Moreover, the use of iron chelation therapy to reduce serum ferritin levels was associated with improved serum glucose or HDL levels [36]. Houschyar et al. studied the effects of phlebotomy and the control of body iron in patients with metabolic syndrome in a randomized, controlled, single-blind clinical trial. The authors concluded that in patients with metabolic syndrome, phlebotomy with a moderate reduction of body iron stores lowered blood pressure and resulted in improvements of markers of cardiovascular risk and glycemic control [37].

The strengths of our study are that the analysis included a large number of subjects (56,053 participants) and thirteen [11-22,31,32] out of the fifteen studies used the NCEP ATP III criteria to diagnose the metabolic syndrome, which is important to reduce possible bias in the results of the individual studies.

Although the odds ratio may overestimate the risk if interpreted as a relative risk, substantial differences between the odds ratio and the relative risk are seen only when the effect size are large and the initial risk is high [38]. In our study, the meta-regression and subgroup analysis did not show difference between odds ratio and hazard ratio (p meta-regression = 0.520) (1.61 vs 1.62), for this reason we combined odds ratio and hazards ratio in pooling analysis.

Another limitation of this meta-analysis is related to the high heterogeneity. However, this elevated heterogeneity was not explained by the study design, type of measure of association, geographic area, ferritin assay technique or study size. Only the use or not of the inflammatory biomarker CRP as control variable and the quality of the studies (≥30 points or < 30 points) were identified as sources of heterogeneity by the subgroup analyses and meta-regression. A possible explanation is that serum ferritin level is an acute-phase reactant and, in the presence of acute or chronic inflammation, may raise several-fold above baseline levels [39,40]. By adjusting by CRP, a proinflammatory biomarker, we controlled for the confounder effect of inflammation, and thus, the association between ferritin levels, as a marker of excessive body iron stores, and the metabolic syndrome was strengthened. We therefore believe it is advisable to use a biomarker of inflammation in the studies investigating the relationship between ferritin and the metabolic syndrome and to ensure that the studies are of high methodological quality.

Another important limitation of this meta-analysis is that cross sectional studies cannot be used to infer a causal role of serum ferritin on the risk of developing metabolic syndrome. Indeed, the mechanisms underlying the relationship between increased ferritin and the metabolic syndrome still need to be clarified. However, we can consider that the results from the twelve cross sectional studies [11-20,22,31], one case–control study [21] are supported by the results of two prospective studies [26,32], conducted in France (944 participants) and in Korea (13,084 participants) [26,32], which established that high levels of serum ferritin preceded the development of metabolic syndrome.

Supporting the biologically plausible pathogenic role of elevated iron, there are other longitudinal studies that noted that high serum ferritin could be a risk factor to develop chronic diseases, especially those related to the metabolic syndrome. Recently, in a Korean cohort on 17,812 healthy men, elevated serum ferritin levels were identified as a predictive factor for obesity [41]. Furthermore, the evidence on a relationship between excess iron and cardiovascular disease has steadily increased over the years [8,42,43]. Moreover, several prospective studies have identified excess iron as a risk factor for T2DM. In the Nurses' Health Study cohort the subjects in the highest quintile had a 2.5 fold higher diabetes-risk than those in the lowest quintile [40]. Similarly, results from the cohort of the European Prospective Investigation into Cancer and Nutrition study (EPIC) also supported the hypothesis that higher iron stores below the level of haemochromatosis are associated with risk of type 2 diabetes [44]. In addition, two recent systematic review and meta-analysis of published prospective studies have confirmed the same relationship [3,4]. Nevertheless, we want to emphasize that our meta-analysis focused on the metabolic syndrome as the primary outcome and that articles on patients with type 2 diabetes mellitus were excluded.

Although several authors have postulated that levels of iron in the upper limit of the normal range are associated with pathological processes [45,46], at present we do not know the cut-off value of serum ferritin concentration that defines the “high level” and that can be considered as a risk factor of metabolic syndrome. The World Health Organization (WHO) has identified levels of ferritin to define iron overload (>200 μg/L for men and >150 μg/L for women) [40-47]. In the studies included in our meta-analysis, we observed that cut-offs of ferritin (≥147 μg/L) in the highest quartile or quintile [11,18,20,26] and the geometric mean of the highest quartile were similar o even lower to the levels used to define iron overload in men. In women the values of ferritin that were associated with metabolic syndrome were 89 μg/L in premenopausal and 212 μg/L in postmenopausal women [11]. These are lower than the values of iron overload defined by the WHO in premenopausal women [11,18,20].

Some studies have observed that premenopausal women have a lower prevalence of hyperferritinemia compared with postmenopausal women, because of iron losses by menstruation and increased consumption during pregnancy [13].

The results of our meta-analysis suggest that the metabolic syndrome could already develop in men and in premenopausal women at ferritin levels that are lower than the WHO cut-offs for iron overload.

High serum ferritin concentrations could potentially be used as a screening biomarker to detect those at risk of developing the metabolic syndrome and those in the early stages of the disease that can still be reversed by targeted preventive measures. For this purpose, data from other countries and races will also be necessary to strengthen our understanding of this relationship and to establish the correct cut-off values of ferritin for each population group.

## Conclusions

In summary, the results of the present meta-analysis indicate that serum high levels of ferritin are independently and positively associated with the metabolic syndrome. Additional prospective studies are needed to confirm if high serum ferritin is a valid biomarker of metabolic syndrome risk, to evaluate the influence of inflammation and to identify pathological cut-off values.

## Abbreviations

CRP: C-reactive protein; EIA: Electrochemiluminescence immunoassay; NCEP ATP III: National cholesterol education program adult treatment panel III criteria; NIA: Nephelometric immunoassay; NR: Not reported; OR: Odds ratio; RIA: Immunoradiometric assay; TIA: Immunoturbidimetric assay.

## Competing interests

The authors declare that they have no competing interests.

## Authors’ contributions

VA-U researched data, interpreted, analyzed the data and wrote the manuscript. GF-M researched data, interpreted, analyzed the data and reviewed the manuscript. RS-A contributed to the discussion and reviewed the manuscript. BM-Y-K contributed to the discussion and reviewed the manuscript. VA reviewed the published data before being included in the analysis, interpreted the data, wrote the manuscript, contributed to the discussion, reviewed the manuscript and is the responsible of all data. All authors read and approved the final manuscript.

## Pre-publication history

The pre-publication history for this paper can be accessed here:

http://www.biomedcentral.com/1471-2458/14/483/prepub
